# The Role of the Paratrigeminal Nucleus in Vagal Afferent Evoked Respiratory Reflexes: A Neuroanatomical and Functional Study in Guinea Pigs

**DOI:** 10.3389/fphys.2015.00378

**Published:** 2015-12-21

**Authors:** Alexandria K. Driessen, Michael J. Farrell, Stuart B. Mazzone, Alice E. McGovern

**Affiliations:** ^1^School of Biomedical Sciences, The University of QueenslandSt Lucia, QLD, Australia; ^2^Monash Biomedicine Discovery Institute and Department of Medical Imaging and Radiation Sciences, Monash UniversityClayton, VIC Australia

**Keywords:** respiratory reflex, vagal ganglia, brainstem, nucleus of the solitary tract, airway afferents, sensory innervation, neuroanatomical tracing

## Abstract

The respiratory tree receives sensory innervation from the jugular and nodose vagal sensory ganglia. Neurons of these ganglia are derived from embryologically distinct origins and as such demonstrate differing molecular, neurochemical and physiological phenotypes. Furthermore, whereas nodose afferent neurons project to the nucleus of the solitary tract (nTS), recent neuroanatomical studies in rats suggest that jugular neurons have their central terminations in the paratrigeminal nucleus (Pa5). In the present study we confirm that guinea pigs demonstrate a comparable distinction between the brainstem terminations of nodose and jugular ganglia afferents. Thus, microinjection of fluorescently conjugated cholera toxin B (CT-B) neural tracers into the caudal nTS and Pa5 resulted in highly specific retrograde labeling of neurons in the nodose and jugular ganglia, respectively. Whereas, nodose neurons more often expressed 160 KD neurofilament proteins and the alpha3 subunit of Na^+^/K^+^ ATPase, significantly more jugular neurons expressed the neuropeptides substance P (SP) and, especially, Calcitonin Gene-Related Peptide (CGRP). Indeed, terminal fibers in the Pa5 compared to the nTS were characterized by their significantly greater expression of CGRP, further supporting the notion that jugular afferents project to trigeminal-related brainstem regions. Electrical stimulation of the guinea pig larynx following selective surgical denervation of the nodose afferent innervation to the larynx (leaving intact the jugular innervation) resulted in stimulus dependent respiratory slowing and eventual apnea. This jugular ganglia neuron mediated response was unaffected by bilateral microinjections of the GABA_A_ agonist muscimol into the nTS, but was abolished by muscimol injected into the Pa5. Taken together these data confirm that jugular and nodose vagal ganglia afferent neurons innervate distinct central circuits and support the notion that multiple peripheral and central pathways mediate sensory responses associated with airway irritations.

## Introduction

The airways and lungs are innervated by heterogeneous populations of vagally-derived sensory neurons, which elicit reflexes and behaviors that contribute to the physiological control of respiration and protect the airways from potentially harmful stimuli (Ricco et al., 1996; Mazzone and Canning, 2002a,b; Undem et al., 2004). There are two main types of airway sensory neurons that can be readily distinguished based on physiological and morphological properties—myelinated large diameter neurons that are exquisitely sensitive to mechanical stimuli and unmyelinated small diameter chemically sensitive neurons that are activated by a range of irritant or pro-inflammatory chemicals. Low threshold mechanosensitive neurons are derived exclusively from the nodose ganglia, while chemosensitive neurons originate in both the nodose and jugular ganglia (Ricco et al., 1996; Undem et al., 2004). Indeed, the ganglionic origin of vagal sensory neurons imparts another source of heterogeneity as the nodose and jugular ganglia have distinct embryological origins and in turn are under the control of distinct transcriptional elements (Nassenstein et al., 2010; D'Autréaux et al., 2011). Thus, the jugular and nodose ganglia are derived from the neural crest and the epibranchial placode, respectively (Nassenstein et al., 2010; D'Autréaux et al., 2011). Consequently, the neurons comprising these ganglia have differing phenotypes, with many jugular neurons containing the neuropeptides substance P (SP) and Calcitonin Gene-Related Peptide (CGRP) and exhibiting a somatic-like phenotype similar to the dorsal root ganglia, while nodose neurons are typically devoid of neuropeptides but express functional purinergic P2X receptors and display a visceral phenotype (Ricco et al., 1996; Undem et al., 2004; Nassenstein et al., 2010; D'Autréaux et al., 2011).

The embryological derivation of vagal sensory neurons not only imparts molecular and functional distinctions to the primary sensory neurons themselves, but also underpins differences in the organization of the primary afferent projections to the brainstem and their resultant higher order central neural circuitry. Thus, we have recently reported in the rat that jugular ganglia neurons predominately project to the paratrigeminal nucleus (Pa5) in the caudal medulla, while the nodose ganglia almost exclusively projects to the nucleus of the solitary tract (nTS; McGovern et al., 2015b). Furthermore, in the same study we employed novel anterograde transynaptic tracing using a conditional herpes simplex virus 1 strain H129 (HSV-1 H129) to show differential higher order airway vagal projections arising from the Pa5 and nTS (McGovern et al., 2015b). This previously unappreciated segregation of jugular and nodose neuronal circuits in the brain raises many questions about the functionality of nodose and jugular ganglia sensory pathways and their respective roles in regulating respiratory reflexes and behaviors.

In the present study we first set out to determine whether nodose and jugular afferent neurons in guinea pigs display differential terminations in the nTS and Pa5, analogous with the observations we have made in the rat. Subsequently, we sought to assess whether the Pa5 plays any role in respiratory reflexes evoked by activation of jugular ganglia derived laryngeal afferent neurons.

## Methods

### Animals

Animal experiments were approved by an institutional Animal Ethics Committee and conducted on adult Dunkin Hartley guinea pigs of either sex (250–350 g, *n* = 25). Animals were housed in a standard environment and given *ad libitum* access to water and food. All efforts were made to ensure that a minimal number of animals were used and that they experienced little discomfort. No notable differences were observed between male and female animals, and as such this comparison did not form a component of the study.

### Conventional neuroanatomical tracing

Dual retrograde tracing, with fluorescently conjugated cholera toxin subunit B (CT-B), was conducted to label the vagal ganglia neurons terminating in the guinea pig nTS and Pa5 using techniques previously reported for rats (McGovern et al., 2015b). In each animal, CT-B_594_ and CT-B_488_ (Molecular Probes, Thermo Fisher Scientific) were microinjected into the nTS and Pa5, respectively. Guinea-pigs (*n* = 6) were anesthetized with isoflurane (2.5% in medical oxygen) via a nose cone and their heads were placed into a stereotaxic frame and flexed at a 45° angle. A midline incision was made through the animal's skin, posterior neck muscles and dura mater to expose the medulla at the level between the occipital bone and C1 vertebra. Using Obex as a reference point (~0.5 mm rostral to the calamus scriptorius), unilateral (*n* = 3 left and *n* = 3 right) microinjections of 2% CT-B (250 nl per injection) were made into the nTS (0.2 mm rostral, 0.2 mm lateral, 0.2 mm dorsal to brain surface) and Pa5 (0.2 mm rostral, 2.8 mm lateral, 0.2 mm dorsal to brain surface) using a calibrated glass micropipette (tip diameter = 30 μm) connected to a pneumatic pressure injector (Pneumatic PicoPump; WPI, Sarasota FL). The glass micropipette was left in the injection site for 5 min after injecting the desired volume to prevent leakage of the tracer onto surrounding tissues. Following microinjections, the midline incision was sutured and animals were allowed to recover for 7 days.

### Tissue harvest and immunohistochemical processing

After 7 days animals were overdosed with sodium pentobarbital (100 mg/kg i.p.) and transcardially perfused with 200 ml of 5% sucrose in 0.1 M PBS (pH 7.4) and 200 ml of 4% paraformaldehyde in 0.1 M PBS (pH 7.4). Brainstems and jugular and nodose vagal ganglia (which are anatomically distinct in guinea pigs) were removed and postfixed overnight in 4% paraformaldehyde, then cryoprotected in 20% sucrose at 4°C. Before further processing, the vagal ganglia were cleaned of excess connective tissue and viewed as wholemounts to confirm the presence of retrogradely traced neurons, following which brainstems and vagal ganglia were frozen in OCT embedding compound and sectioned on a Leica CM 1850 UV cryostat. The jugular and nodose vagal ganglia were sectioned at 12 μm and thaw mounted onto gelatin-coated slides across six sets while the brainstems were sectioned at 50 μm and collected serially in 0.1 M PBS.

Sections of brainstem (free floating) and vagal ganglia (slide-mounted) were blocked with 10% goat serum in 0.1 M PBS for 1 h at room temperature, following which they were incubated overnight in the primary antibody of interest (diluted in 2% goat serum and 0.3% Triton-X 100 in 0.1 M PBS). For vagal ganglia, vesicular glutamate transporter 1 (VGLUT1), Neurofilament 160 kD, α3- Na^+^/K^+^ ATPase, CGRP, and SP immunohistochemistry was performed each on separate sets of slides (see Table 1 for details) as our previous studies have shown that these markers help differentiate distinct subsets of vagal afferents (Mazzone and McGovern, 2008). For brainstems, every alternate section (i.e., every 100 μm) was immunostained for the calcium binding protein calbindin to provide morphological detail. After several washes, tissues were incubated in the relevant secondary antibody (Table 1) for an hour at room temperature. Sections labeled with biotinylated secondary antibodies were further incubated with streptavidin conjugated to Alexa Fluor 350, (1:200 dilution; Molecular Probes, Thermo Fisher Scientific). After immunostaining brainstem sections were mounted onto gelatin-coated slides and all slides were then coverslipped with an antifade mounting media (Fluroshield, Sigma Aldrich). Brainstems and vagal ganglia were also removed from four separate perfused fixed animals (i.e., containing no CT-B tracing) and sectioned as described above. These tissues were subsequently immunostained for both CGRP and SP and used for quantifying the relative neuropeptide expression in the two ganglia (jugular vs. nodose) and two brainstem (nTS vs. Pa5) regions of interest. All tissues were viewed using an Olympus BX51 fluorescent microscope equipped with appropriate filters, and images were captured using an Olympus DP72 camera. Representative images were assembled in Adobe Photoshop CS6.

**Table 1:** **Primary and secondary antibody specifications**.

**Antibody**	**Supplier**	**Host**	**Dilution**
**PRIMARY ANTIBODIES**			
Calbindin D-28k	Swant	Rabbit	1:1000
Calcitonin Gene Related Peptide [4901]	Abcam	Mouse	1:1000
Substance P (SP)	Millipore	Rat	1:500
Neurofilament 160 kDa, NN18	Millipore	Mouse	1:1000
VGLUT1	Synaptic Systems	Rabbit	1:500
α3- Na^+^/K^+^ ATPase, XVIF9-G10	Enzo Life Sciences	Mouse	1:200
**SECONDARY ANTIBODIES**			
Biotinylated Anti-Rabbit IgG	Vector Labs	Goat	1:500
Biotinylated Anti-Mouse IgG	Vector Labs	Goat	1:500
Biotinylated Anti-Rat IgG	Vector Labs	Goat	1:500
Anti-Mouse IgG (H+L) Alexa Fluor 594	Life Technologies	Donkey	1:500
Anti-Rat IgG (H+L) Alexa Fluor 488	Life Technologies	Goat	1:500

### Histology and tracing data analysis

Retrogradely traced CT-B neurons were quantified in the jugular and nodose vagal ganglia sections (ipsilateral to the injection site) by counting CT-B_594_ and CT-B_488_ labeled cells with clearly identifiable nuclei in each ganglia section collected. Total CT-B positive cell counts for the nodose or jugular ganglia were calculated by summing the cell counts obtained for each animal and then averaging these sums across animals. CT-B traced cells were further classified based on their co-expression of the selected immunomarkers and the percentages of CT-B and immunomarker double positive cells per tissue were calculated. For somal size analysis, stored digital images of CT-B labeled neurons were imported into Image-J software (National Institutes of Health, Bethesda, Maryland, USA) and cell perimeters were manually traced on screen using a calibrated drawing tool. Only cells with an identifiable nucleus were measured in order to increase the likelihood that perimeters were a reflection of true somal size. A minimum of 100 neurons were measured from both the jugular and nodose ganglia. There were no notable differences between experiments conducted with CT-B injections into the left vs. right brainstem (*n* = 3 for each) and as such the ganglia cell count data were pooled (*n* = 6).

The brainstem injection sites of CT-B_594_ and CT-B_488_ in the nTS and Pa5, respectively were viewed in serial sections 100 μm apart to calculate the rostrocaudal spread of injectate both relative to the identified injection site and the anatomical landmark Obex. Photomicrographs of brainstem sections immunostained for SP and CGRP were collected at the same magnification and identical exposure for quantification of immunostaining intensity. Images were imported unmodified into Image-J and a pixel density analyses was performed over a standardized area in each image of the nTS and Pa5 (the area dimensions of the density tool equalled 428 μm in the mediolateral direction and 284 μm in the dorsoventral direction). A region of no immunostaining within the same image was always quantified to further normalize the data to the level of background pixel density. Total pixel density was defined as the sum of density measurements for a given region obtained from serial sections that approximately spanned the region of CT-B injectate spread, calculated above. Such measurements were calculated for CGRP and SP in the nTS and Pa5 from an equal number of sections and data were background corrected and averaged across animals. All histological data are presented as the mean ± SEM and statistical comparisons were made using an unpaired Students *t*-test with significance set at *p* ≤ 0.05.

### Physiology

Guinea pigs (*n* = 17) were anesthetized with urethane (1.5 g/kg i.p.), the level of which was tested by assessing the animals withdrawal reflex. Animals were placed in a supine position on a thermostatically controlled heating pad. An incision was made along the ventral surface of the neck and the muscles overlaying the trachea were retracted to expose the larynx, trachea and underlying nerves and blood vessels. The left carotid artery was cannulated using polyethylene tubing (internal diameter = 0.5 mm, outer diameter = 0.9 mm) attached to a pressure transducer filled with heparinized saline (50 U/ml) to measure arterial blood pressure (ABP) and heart rate (HR). In addition, the distal extrathoracic trachea was cannulated. The cannula was connected via a sideport to a pressure transducer to measure the tracheal pressure (T_P_) changes associated with spontaneous respiration. Output from the pressure transducers were filtered and amplified (NeuroLog Systems, Digitimer, Hertfordshire, UK), digitized (Micro1401 A-D converter, CED, Cambridge, UK) and recorded using Spike II software (CED, Cambridge, UK) for offline analysis.

**Figure 1:**
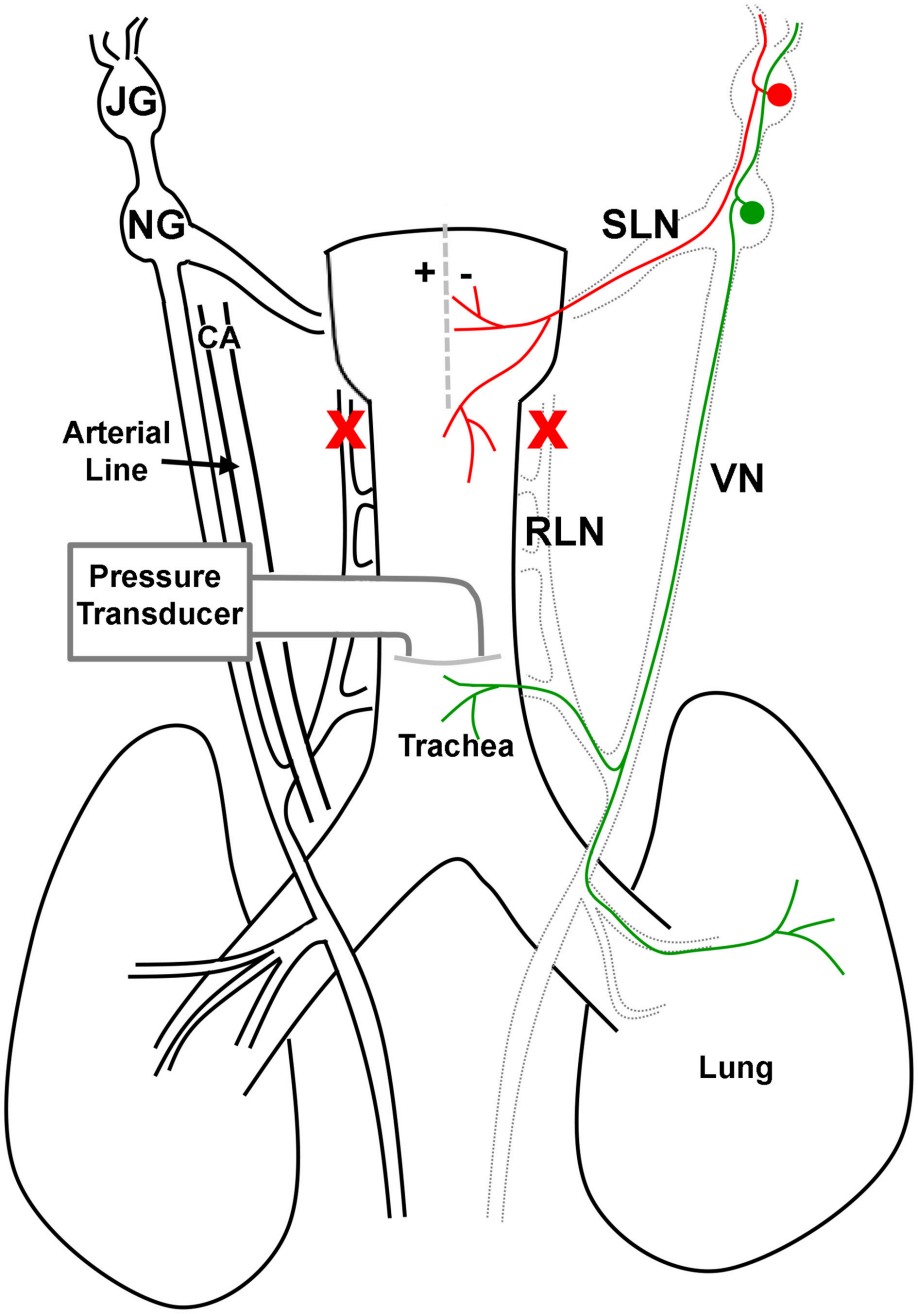
**Schematic representation of the vagal innervation of the guinea pig airways and preparation used in this study**. In the guinea pig, the larynx is innervated by jugular (JG) derived afferents (red) traveling primarily in the superior laryngeal nerves (SLN) and nodose (NG) derived afferents (green) principally traveling in the recurrent laryngeal nerves (RLN). In spontaneously breathing anesthetized guinea pigs, the distal-most portion of the trachea was cannulated and connected to a pressure transducer in order to measure tracheal pressure. In addition, the left carotid artery (CA) was cannulated and attached to a pressure transducer to measure arterial blood pressure and heart rate. The RLNs were cut bilaterally (red crosses) to remove the nodose afferent innervation to the larynx, leaving intact the jugular-superior laryngeal nerve pathway. A midline incision through the laryngeal thyroid cartilage (dashed line) was then made to expose the mucosal surface for the placement of a custom in-house built platinum bipolar stimulating electrode (represented by + −). Electrical stimulation of the larynx was used to evoke reflex changes in respiration.

In the guinea pig, the larynx is innervated by jugular-derived afferents traveling principally in the superior laryngeal nerves and nodose-derived afferents principally traveling in the recurrent laryngeal nerves (RLNs; Canning et al., 2004). Accordingly, both RLNs were gently blunt dissected from the larynx and trachea and subsequently cut in order to selectively denervate the nodose afferent innervation of the larynx, leaving intact the jugular-superior laryngeal nerve pathway (see Figure 1 for more details). A midline incision through the laryngeal thyroid cartilage was then made to expose the mucosal surface for the placement of a custom in-house built platinum bipolar stimulating electrode.

After a 20 min stabilization period, electrical stimulation of the larynx was performed (model s48, Grass Instruments) by delivering increasing voltages (0.1–10 V) to the larynx at a constant stimulating frequency (32 Hz), pulse durations (1 ms) and train duration (10 s), expected to activate mucosal and deeper laryngeal tissue afferents. The voltage that evoked a reproducible maximum respiratory response (defined as the optimum voltage, which on average = 7.6 ± 0.4 V) was then used to assess the frequency (1–32 Hz) dependency of the evoked response under the same conditions. At the completion of the electrical stimulations, animals were placed into a stereotaxic frame and the brainstem exposed. The GABA_A_ agonist, muscimol (20 ng in 150 nl) or saline (vehicle 150 nl) was then microinjected bilaterally into either the nTS (*n* = 4 muscimol, *n* = 3 vehicle) or Pa5 (*n* = 4 muscimol, *n* = 4 vehicle) following the same protocol as described above. Animals were immediately returned to a supine position and the stimulating electrode was replaced onto the laryngeal surface in order to repeat the frequency response stimulations at the predefined optimal stimulating voltage. Electrical stimulation following microinjection of either muscimol or vehicle was completed within a period of 30 min. In additional animals the superior laryngeal nerves (SLNs) and the RLNs were cut bilaterally to completely denervate the vagal innervation to the larynx.

Physiological data at each stimulation frequency were calculated from the chart recordings and compared to baseline data (defined as the 60 s period prior to the commencement of the frequency response curve). For respiration, breathing frequency was calculated over the 10 s period of the stimulation and multiplied by six to obtain the equivalent breaths/minute. Mean arterial blood pressure responses were calculated at the peak effect during stimulation and compared to baseline data. All physiological data are represented as the mean ± SEM and differences between groups were tested using a Two-way ANOVA with significance set at *p* ≤ 0.05. In addition, the evoked the maximum physiological effect (Emax) for each experiment was determined prior to vehicle or muscimol injection (defined as the maximum response evoked regardless of the stimulation parameters) and compared to the same stimulation parameters after microinjection of vehicle or muscimol into the nTS or Pa5. These data were compared using a paired *t*-test with significance set at *p* ≤ 0.05.

## Results

### Retrograde tracing and immunohistochemical characterization of vagal sensory neurons

**Figure 2:**
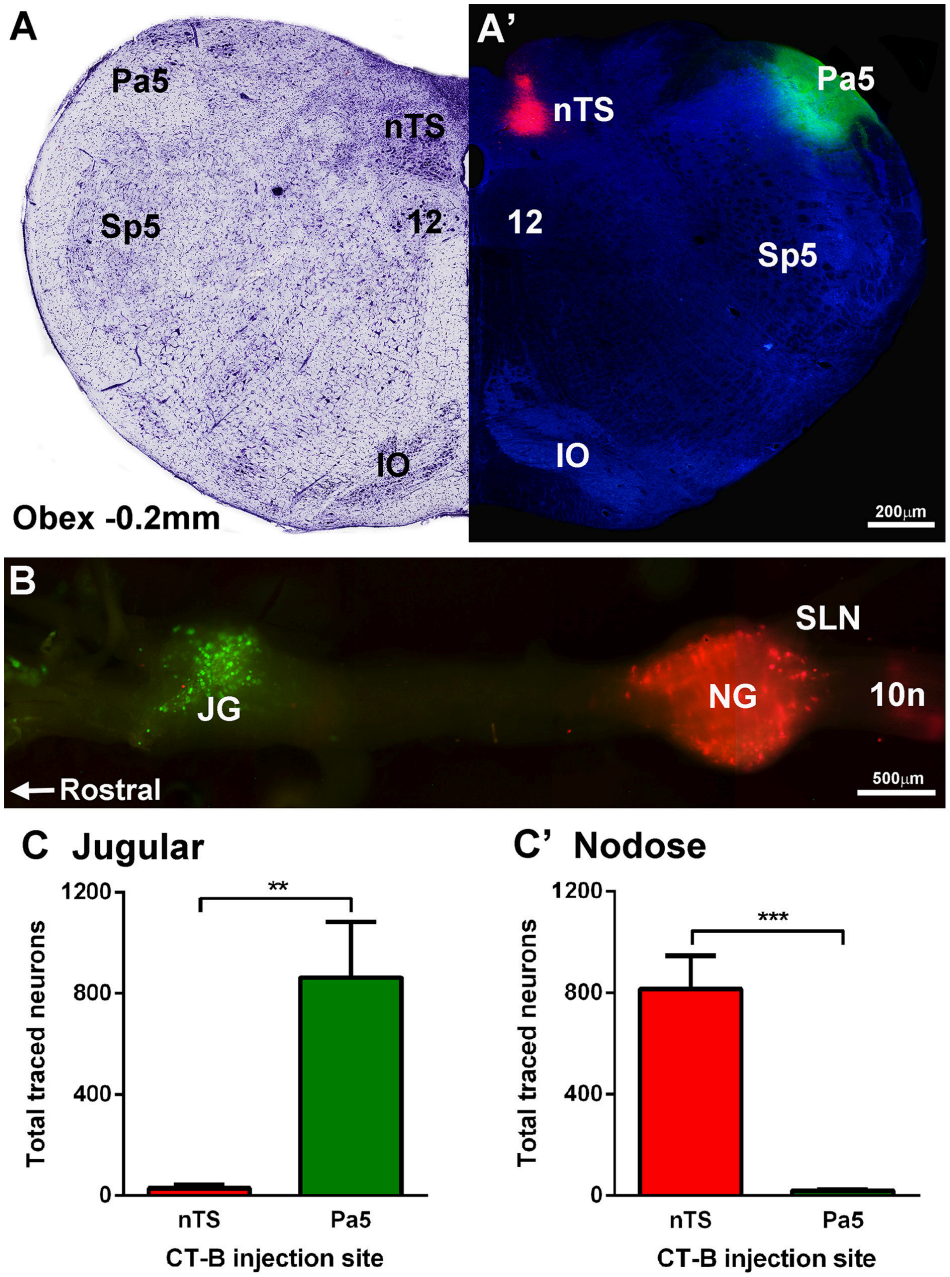
**Differential terminations of jugular and nodose vagal ganglia neurons in the paratrigeminal nucleus (Pa5) and nucleus of the solitary tract (nTS). (A,A′)** Example photomicrograph and corresponding Nissl staining of a caudal brainstem section showing the Pa5 and nTS locations for microinjection of cholera toxin subunit-b (CT-B) tracers conjugated with 488 (green) or 594 (red) fluorophores, respectively. **(B)** Fluorescent photomicrograph of a wholemount preparation of the guinea pig vagal ganglia showing neuronal soma retrogradely labeled with CT-B from the Pa5 (green) and nTS (red). **(C)** Quantitative cell counts performed on serial ganglia sections, demonstrating that Pa5 projecting neurons reside in the jugular ganglia (JG) whereas **(C**′**)** nTS projecting neurons are located within the nodose ganglia (NG). Data represent the mean ± SEM number of neurons quantified from a minimum of 10 tissue sections of vagal ganglia obtained from *n* = 6 separate dual tracing experiments. ^**^P ≤ 0.01 and ^***^P ≤ 0.001, significantly different pairwise comparison. Additional abbreviations: 10n, vagus nerve; 12, hypoglossal nucleus; IO, inferior olives; SLN, superior laryngeal nerve; Sp5, spinal trigeminal nucleus; sp5, spinal trigeminal tract.

Dual microinjection of fluorescent CT-B_488_ and CT-B_594_ conjugates successfully encompassed the target regions in all animals (Figures 2A,A′). The rostrocaudal spread of injectate within each nucleus was similar (nTS, 0.94 ± 0.11 mm and Pa5, 1.68 ± 0.25 mm) with the center of the injection located just rostral to Obex (nTS, 0.62 ± 0.16 mm and Pa5 0.48 ± 0.19 mm). The mediolateral spread never exceeded 0.2 mm (nTS) and 0.3 mm (Pa5) in any animal and as such there was never any overlap between the different injectates delivered to the two brainstem nuclei. Importantly, retrogradely labeled neurons were clearly visible within the jugular and nodose vagal ganglia, albeit with a distinct topographical arrangement, as shown in a representative wholemount image (Figure 2B). Thus, there was a distinct separation of neurons retrogradely labeled from the Pa5 vs. the nTS in the jugular and nodose ganglia, respectively. Quantitative cell counts on tissue sections, confirmed that the jugular ganglion had significantly more neurons retrogradely labeled from the Pa5 compared to the nTS (*p* = 0.0037; Figure 2C). On the other hand, the nodose ganglion had significantly more retrogradely labeled neurons from the nTS compared to the Pa5 (*p* = 0.0001; Figure 2C′). There were no systematic differences between ganglia counts collected from animals receiving right vs. left brainstem injections (not shown).

**Figure 3:**
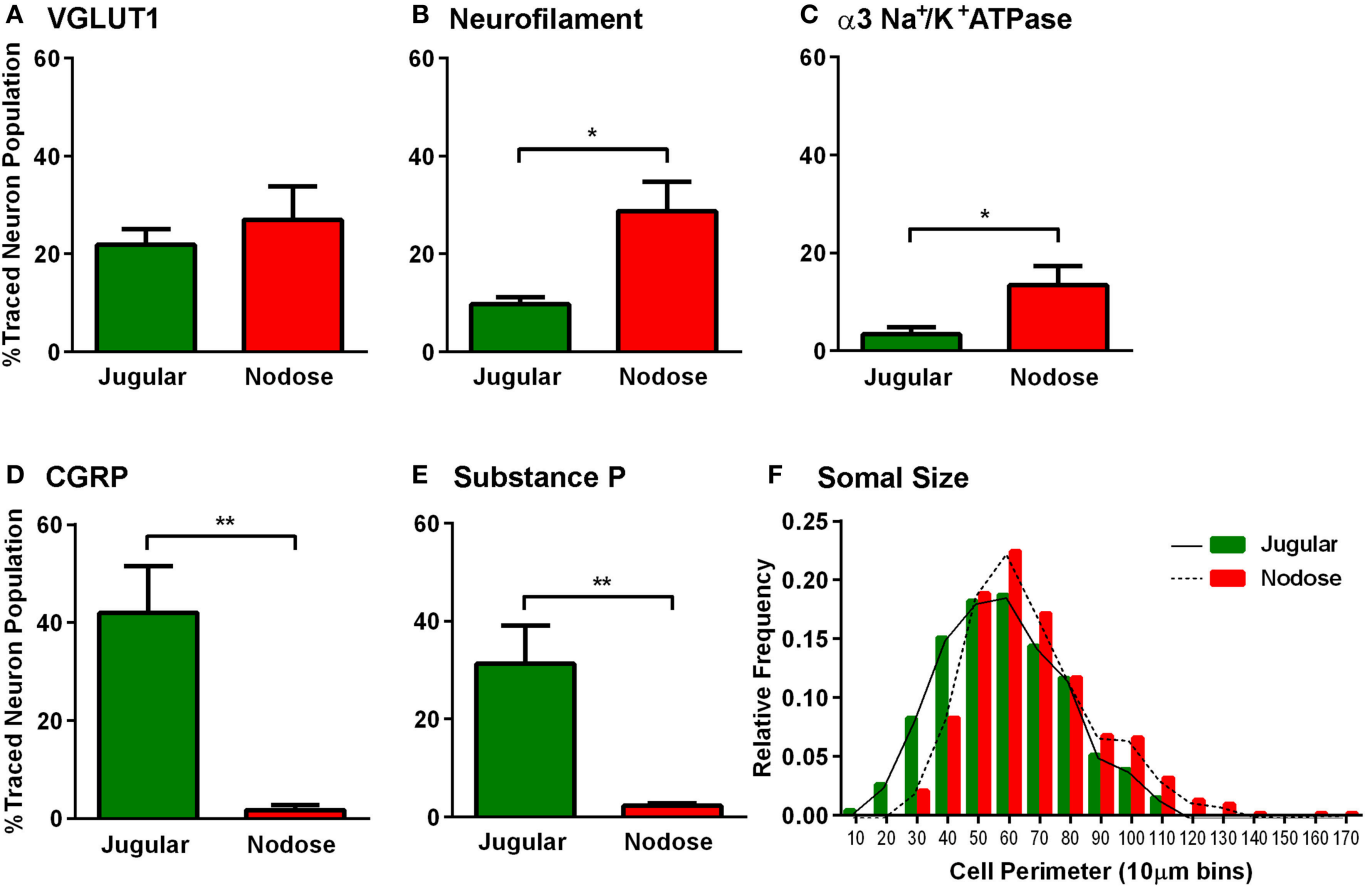
**Characterization of the vagal sensory neurons projecting to the nucleus of the solitary tract and paratrigeminal nucleus**. Neurons retrogradely traced from either the nucleus of the solitary tract (red) or the paratrigeminal nucleus (green) were characterized based on their expression of several immunomarkers. Bar graphs demonstrate that **(A)** VGLUT1 is common to both nTS and Pa5 projecting vagal sensory neurons. More nTS projecting vagal neurons express **(B)** neurofilament and **(C)** α3 Na^+^/K^+^ ATPase. More paratrigeminal projecting neurons express the neuropeptides **(D)** CGRP and **(E)** SP. Data represent the mean ± SEM percentage of traced neurons expressing each immunomarker quantified from a minimum of 10 tissue sections of vagal ganglia obtained from *n* = 6 separate dual tracing experiments. ^*^P ≤ 0.05 and ^**^P ≤ 0.001 significantly different pairwise comparison. **(F)** The histogram shows that the distributions of traced neuron somal sizes are comparable between both nTS and Pa5 projecting vagal sensory neurons. These data represent pooled somal sizes of at least 100 neurons obtained from *n* = 6 separate experiments. The relative frequency is the proportion of the total pooled population with a given somal size.

**Figure 4:**
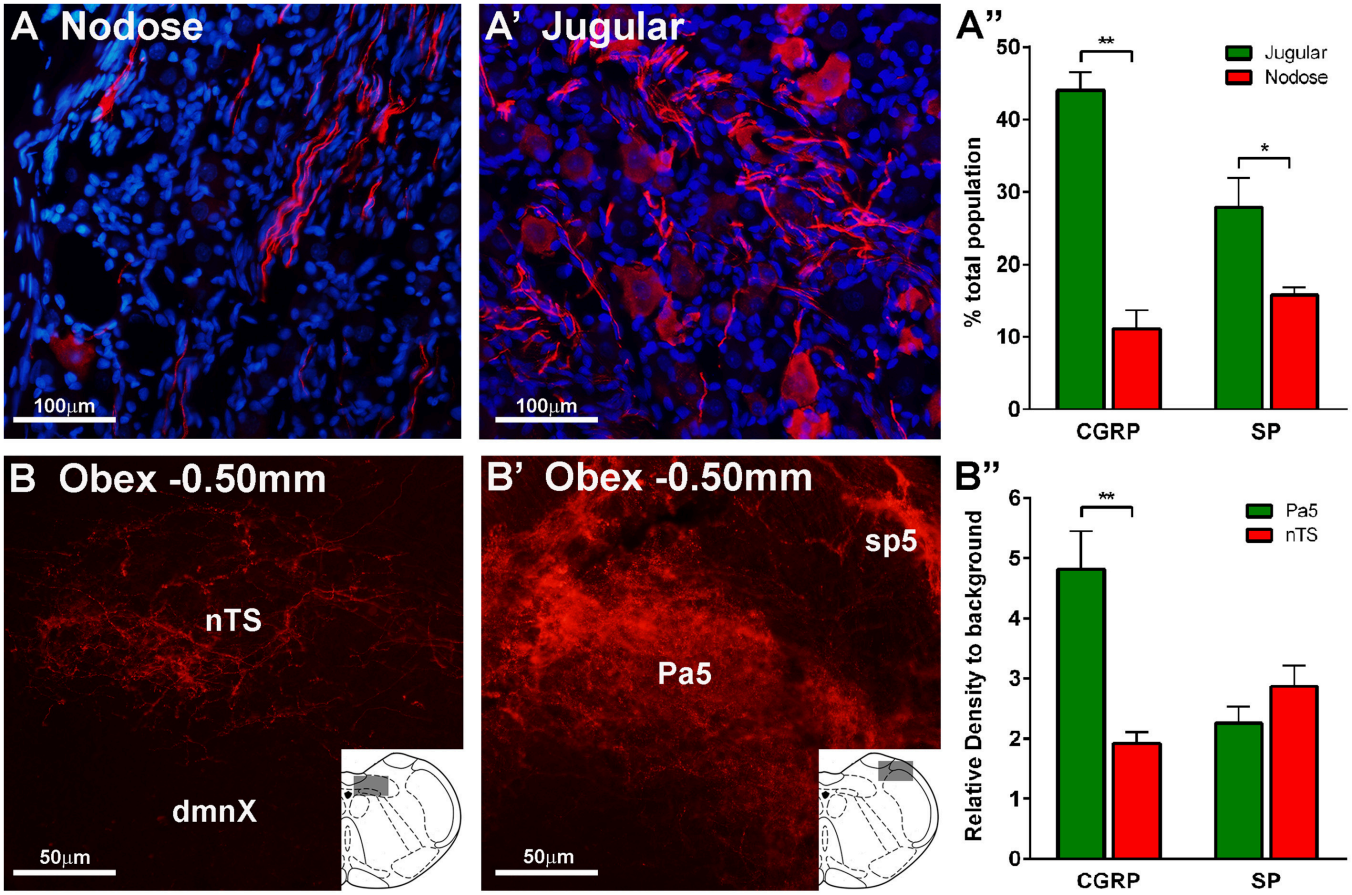
**Expression of neuropeptides calcitonin gene-related peptide (CGRP) and substance P (SP) in the vagal sensory ganglia and brainstem**. Representative photomicrographs showing differential expression of CGRP in the **(A)** nodose ganglia, **(A**′**)** jugular ganglia, **(B)** nucleus of the solitary tract (nTS), and **(B**′**)** paratrigeminal nucleus (Pa5). Bar charts show quantitative analysis of CGRP and SP expression in **(A**″**)** vagal ganglia and **(B**″**)** brainstem. Neuropeptide expression is more abundant in the jugular than the nodose ganglia. SP expression in the brainstem is similar in the nTS and Pa5, whereas expression of CGRP is significantly higher in the Pa5 compared to the nTS. Data represent the mean ± SEM number neurons **(A**″**)** or intensity of immunostaining **(B**″**)** quantified from a minimum of 10 tissue sections of vagal ganglia obtained from *n* = 6 separate experiments. ^*^P ≤ 0.05 and ^**^P ≤ 0.01 significantly different pairwise comparison. Additional abbreviations: dmnX, dorsal motor nucleus of the vagus nerve; sp5, spinal trigeminal tract.

Further analyses of the retrogradely traced vagal neurons revealed differences in their immunohistochemical profiles. Thus, there were significantly more peptidergic (CGRP and SP expressing) CT-B labeled neurons (presumably nociceptors) in the jugular ganglia compared to the nodose ganglia (*p* = 0.002, *p* = 0.004 respectively, Figures 3D,E). Conversely, the number of CT-B traced neurons expressing neurofilament and α3 Na^+^/K^+^ ATPase, markers of myelinated neurons including low threshold mechanoreceptors, was higher in the nodose compared to the jugular ganglia (*p* = 0.012, *p* = 0.038 respectively, Figures 3B,C). However, VGLUT1 also expressed by myelinated nodose neurons (Mazzone and McGovern, 2008) was expressed by a similar number of CT-B labeled jugular and nodose neurons (Figure 3A). Quantification of traced cell perimeters confirmed that CT-B labeled a wide range of cell types from the nTS and Pa5 (encompassing the known distribution of somal sizes in the guinea pig vagal ganglia; (Mazzone and McGovern, 2008) and indeed there was no difference in the size distribution of traced cells between the two ganglia (Figure 3F), suggesting that our tracing experiments were not skewed toward a certain afferent subtype. Across the total population of vagal ganglia neurons there were significantly more neurons within the jugular ganglia compared to the nodose that expressed the neuropeptides CGRP and substance P (*p* = 0.00004 and *p* = 0.025 respectively, Figures 4A,A′,A″). Quantification of immunohistochemical staining intensity within the brainstem revealed significantly higher levels of CGRP, but not SP, present in the Pa5 compared to the nTS (*p* = 0.002, Figures 4B,B′,B″).

### Functional studies

**Figure 5:**
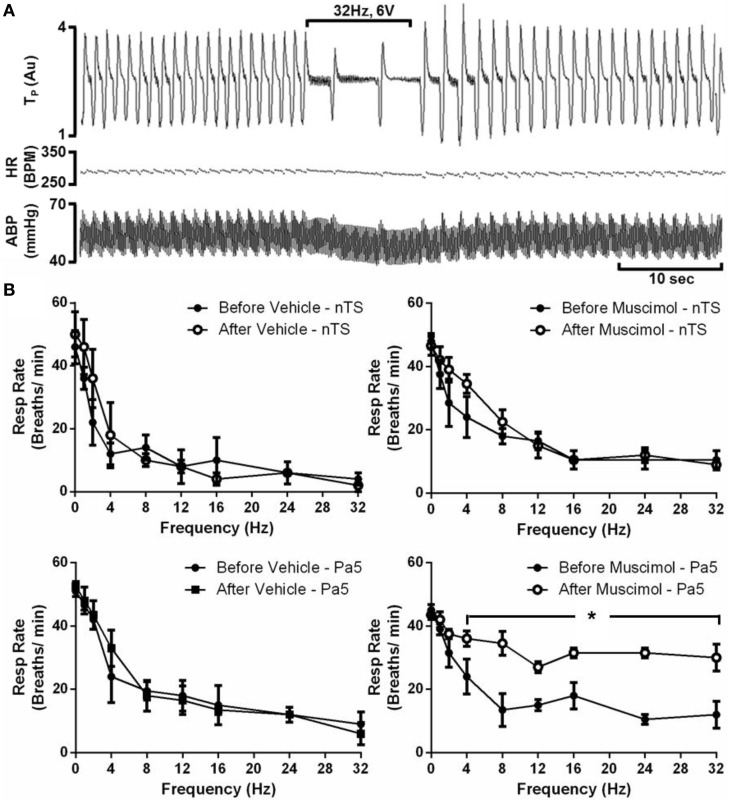
**Laryngeal evoked respiratory reflexes are dependent upon a jugular—paratrigeminal neural circuit**. **(A)** Representative physiological recording showing the slowing of respiration and reduction in blood pressure, without any change in heart rate, during electrical stimulation of the larynx in RLN transected animals. **(B)** Mean frequency-dependent effects on (Resp) rate before and after bilateral microinjection of vehicle or muscimol into the nucleus of the solitary tract (nTS) or paratrigeminal nucleus (Pa5). Each graph is the mean ± SEM of four animals ^*^P ≤ 0.05 significantly different responses at 4–32 Hz compared to the corresponding response before injection. Abbreviations: ABP, arterial blood pressure; AU, arbitrary units; BPM, beats per minute; HR, heart rate; TP, tracheal pressure.

Electrical stimulation of the larynx, in animals with RLN transections, evoked respiratory slowing resulting in complete apnea for the stimulus duration at higher stimulus voltages (above 8 volts). Rarely (less than 5% of occasions) animals coughed in response to electrical stimulation, consistent with the notion that the RLN transection removes most of the nodose afferent innervation of the larynx (Canning et al., 2004). At optimum stimulus intensities, increasing stimulus frequency evoked a frequency dependent reduction in respiratory rate as well as a modest decrease in blood pressure, while no effect was seen in heart rate (Figures 5A,B; Table 2). Microinjection of saline into the nTS or the Pa5 did not alter the frequency dependent fall in respiratory rate (Figure 5B) or blood pressure (Table 2). Bilateral microinjection of muscimol into the nTS did not alter basal respiration (Figure 5B), but caused mean arterial blood pressure to significantly decrease on average from 61.2 ± 4.18 to 44.8 ± 4.66 mmHg (*p* = 0.0051). However, muscimol in the nTS did not alter the reduction in respiratory rate evoked by laryngeal stimulation in RLN transected rats (Figure 5; Table 2). The same dose of muscimol microinjected into the Pa5 neither altered basal respiratory rate (Figure 5B) nor blood pressure (63.6 ± 4.0 vs. 56.8 ± 2.8 mmHg before and after muscimol injection) but almost abolished the respiratory and blood pressure responses associated with laryngeal stimulation (Figure 5; Table 2). Thus, the Emax for the respiratory slowing did not differ between pre (mean = 10.5 ± 2.87 breaths/min) and post (mean = 9.0 ± 1.73 breaths/min) muscimol administration to the nTS (Table 2) but was significantly different between pre (mean = 6.0 ± 3.46 breaths/min) and post (mean = 25.5 ± 2.87 breaths/min) muscimol administration to the Pa5 (*p* = 0.001, Table 2). In 2 animals, bilateral denervation of both RLNs and SLNs completely abolished changes in respiration evoked by electrical stimulation of the larynx, confirming the vagal dependency of the evoked response (data not shown).

**Table 2:** **Maximum laryngeal stimulation-evoked respiratory and cardiovascular responses**.

**Treatment groups**	**Respiration (breaths/min)**		**Blood Pressure (mmHg)**		**Heart Rate (bpm)**	
	**Emax (Mean ± SEM)**	***p*-value**	**Emax (Mean ± SEM)**	***p*-value**	**Emax (Mean ± SEM)**	***p*-value**
nTS before vehicle/after vehicle (*n* = 3)	4.0 ± 2.0/2.0 ± 2.0	0.667	43.5 ± 1.57/49.8 ± 3.47	0.324	281.7 ± 6.00/282.3 ± 8.69	0.843
Pa5 before vehicle/after vehicle (*n* = 4)	9.0 ± 3.87/6.0 ± 3.46	0.181	48.3 ± 7.11/48.6 ± 5.61	0.884	286.5 ± 26.18/302.7 ± 18.10	0.216
nTS before muscimol/after muscimol (*n* = 4)	10.5 ± 2.87/9.0 ± 1.73	0.391	51.5 ± 4.64/38.3 ± 5.15	0.052	286.7 ± 15.60/277.7 ± 4 0.27	0.751
Pa5 before muscimol/after muscimol (*n* = 4)	6.0 ± 3.46/25.5 ± 2.87	0.001^*^	53.5 ± 4.79/50.5 ± 1.93	0.577	316.3 ± 10.65/309.5 ± 8.13	0.111

Baseline (prior to any treatments or stimulation) respiratory rate (breaths/min) = 46.93 ± 1.69; blood pressure (mmHg) = 60.34 ± 2.19; heart rate (bpm) = 301.93 ± 8.06.

* *Significantly different compared to before treatment*.

## Discussion

We have previously reported our investigations into the organization of airway sensory neural circuitry in the rat brain, showing that anatomically distinct central circuits arise from nodose and jugular vagal ganglia airway afferents. Thus, in rats, nodose and jugular afferent neurons specifically innervate second order neurons in the nTS and Pa5, respectively reflecting a previously unrecognized anatomical divergence of vagal afferent inputs to the brainstem (McGovern et al., 2015b). Importantly, while the role of vagal afferent processing in the nTS has been investigated in significant detail, the functionality of the jugular-Pa5 pathway with respect to responses evoked by airway irritation has not been previously explored. In the present study we first confirmed that this anatomical segregation of vagal afferent circuitry in the brainstem is similarly expressed in the guinea pig, and is therefore not peculiar to the rodent vagal system. Indeed, whilst heterogeneous nodose and jugular afferent neurons project to both brainstem integration sites, jugular afferents can be readily differentiated from nodose afferents both in the sensory ganglia and in the brainstem by the expression of the neuropeptide CGRP. Secondly, we demonstrate that jugular ganglia afferent mediated respiratory responses following laryngeal stimulation are abolished by inhibition of the Pa5 and not the nTS. Collectively, these data provide support for the existence of two parallel yet distinct airway vagal sensory processing pathways that likely differentially contribute to respiratory responses evoked by airway irritant stimuli.

### Evidence that vagal ganglia afferents have multiple distinct termination sites in the brainstem

A variety of studies confirm that airway sensory neurons originate in both the nodose and jugular vagal ganglia and that the distinct embryological origins of these ganglia confer the constituent neurons with meaningful differences in their functional, neurochemical, neuroanatomical and molecular expression characteristics (Ricco et al., 1996; Kollarik and Undem, 2002; Undem et al., 2004; Kwong et al., 2008; Nassenstein et al., 2010; D'Autréaux et al., 2011; McGovern et al., 2015a). Indeed, investigations of the development of the cranial ganglia have identified transcriptional elements such as Phox2b and Runx1 that determine a visceral vs. somatic fate of ganglia neurons. Specifically, nodose neurons represent visceral afferents inasmuch as they develop from an epibranchial placode lineage that is dependent upon transient developmental Phox2b expression, whereas jugular neurons, like the spinal dorsal root ganglia, reflect somatic neurons derived from neural crest cells under the control of Runx1 (Nassenstein et al., 2010; D'Autréaux et al., 2011). This fundamental source of afferent heterogeneity may in turn underpin differences in the organization of nodose and jugular afferent terminals in the brainstem which show preferential innervation of the nTS and trigeminal nuclei, respectively. Thus, our lab recently showed in the rat that CT-B microinjections into the region of the Pa5 resulted in the retrograde labeling of jugular, but not nodose neurons, whereas a comparable injection into a caudal lateral site in the nTS resulted in retrograde labeling of the nodose, but not jugular ganglia neurons (McGovern et al., 2015b). Furthermore, in several studies we have shown that both upper and lower airway injections of the anterograde transynaptic neurotropic virus HSV-1 H129 results in time-dependent dorsolateral nTS and Pa5 infections that develop with comparable kinetics indicative of two distinct vagal pathways terminating in the brainstem (McGovern et al., 2015a).

In the present study, CT-B injections into the caudal lateral nTS and Pa5 of the guinea pig resulted in the selective retrograde labeling of nodose and jugular afferent neurons, respectively. Indeed, the anatomical specificity of the CT-B labeling from the two injection sites was extraordinary, with few nTS-projecting jugular or Pa5-projecting nodose neurons identified. Although our pathway tracing was not airway afferent specific (i.e., the peripheral terminals of the retrogradely labeled sensory neurons were not identified) the striking segregation of nodose and jugular afferent terminations in brainstem regions that receive airway vagal afferent input (McGovern et al., 2015a,b) would argue that the data can be confidently extrapolated to conclude that airway vagal afferents share this termination pattern. Thus these results, along with our previous studies, argue for parallel airway sensory processing pathways involving the jugular ganglia projecting to the Pa5 and the nodose ganglia projecting to the nTS. Furthermore, the data suggest that this anatomical organization is conserved across species, at least for the specific brainstem loci targeted by the current microinjection strategy. However, we cannot rule out if other nTS sites receive jugular afferent inputs, such as the commissural nTS from which some retrogradely traced jugular neurons have been identified (Mazzone and Canning, 2002a). Even in our own studies we see a small but consistent population of jugular projections to the nTS in both the rat (McGovern et al., 2015b) and guinea pig (present study). The reason why some jugular neurons may differ to others with respect to their central projections is presently unclear.

### CGRP expression distinguishes jugular and nodose sensory neurons in the vagal ganglia and brainstem

The present neuroanatomical studies were not intended to differentiate between vagal ganglia neurons terminating in different peripheral tissues. Nevertheless, important insight into the heterogeneity of vagal afferent neurons can gained from such studies. Thus, a heterogeneous population of sensory neurons innervates the airways, delineated by their vagal ganglia origin and neurochemical profile. For example, in the large airways (larynx, trachea, and main bronchi) the majority of nodose derived neurons express medium or large molecular weight neurofilament proteins, the α3 subunit of Na^+^/K^+^ ATPase and VGlut1, while many of the large airway projecting neurons derived from the jugular ganglia are characterized by the expression of the neuropeptides SP and CGRP (Undem et al., 2004; Mazzone and McGovern, 2008). Consistent with this, we found that more of the nodose neurons that were retrogradely labeled from the nTS expressed neurofilament and α3 Na^+^/K^+^ ATPase (but not vGlut1) compared to jugular neurons labeled from the Pa5. Whereas, retrogradely traced jugular neurons were more often immunoreactive for CGRP and SP than those in the nodose ganglia. This differential expression is unlikely due to selectivity of the CT-B neural tracer for different subsets of nodose and jugular neurons, as the profile of somal sizes of traced neurons was not different between ganglia.

We further reasoned that the generalized differential molecular expression noted in the ganglia may be similarly conserved in the central terminals of the vagal afferents innervating the nTS and Pa5. Given that neurofilaments and α3 Na^+^/K^+^ ATPase are widely expressed in central neural tissue (Trojanowski et al., 1986; McGrail et al., 1991), we restricted this investigation to the neuropeptides that readily differentiate nodose from jugular neurons. Consistent with previous studies in other species (Franco-Cereceda et al., 1987; Sugimoto et al., 1997) and with the notion that jugular ganglia neurons specifically project to the Pa5 (present study), we noted dense CGRP staining in the Pa5 that was significantly greater than that in the nTS. However, it is unlikely that vagal ganglia neurons represent the only afferent source of CGRP to the Pa5 as trigeminal ganglia neurons also express the neuropeptide (Eberhardt et al., 2008). By contrast, the expression of SP was not significantly different between the two brainstem nuclei. Indeed SP staining intensity in the nTS appeared disproportionately high relative to the number of SP-positive neurons identified in the nodose ganglia, perhaps reflecting alternative origins of SP terminals in the nTS. Intrinsic neurons or the terminals of nTS inputs that arise from other peripheral or central neuronal populations can express SP (South and Ritter, 1986; Gallagher et al., 1992), which may explain why vagal denervation does not abolish SP immunoreactivity in the nTS (Gillis et al., 1980; Kawai et al., 1989). Given these findings it is tempting to speculate that CGRP may play an important role in the integration and/or sensitization of jugular ganglia vagal afferent responses in the Pa5. Indeed, CGRP has been implicated in trigeminal nociception, orofacial pain, algesic sensitization and migraines (Devesa et al., 2014; Bigal et al., 2015; Romero-Reyes et al., 2015), and a comparable pro-nociceptive role for the neuropeptide in vagal-evoked responses seems plausible given the somatic nature of the juglar-Pa5 pathway. Nevertheless, this putative role for CGRP awaits further exploration.

### A jugular ganglia-Pa5 neural circuit contributes to laryngeal evoked respiratory reflexes

Electrical, mechanical or chemical stimulation of the guinea pig larynx has been shown to evoke a variety of respiratory and cardiovascular responses, including cough, apnea/respiratory slowing, bronchoconstriction, and hypotension (Mazzone and Canning, 2002a,b; Canning et al., 2004; Chou et al., 2008). Electrophysiological and neuroanatomical tracing studies have confirmed that the larynx receives both nodose and jugular afferent innervation, although the route via which nodose and jugular axons reach the larynx is not uniform (Canning et al., 2004; Mazzone et al., 2009). In electrophysiological mapping studies 88% of nodose ganglia neurons (almost all are low threshold mechanoreceptors) innervate the rostral guinea pig trachea and larynx via the RLNs, while 84% of jugular ganglia neurons (all C- and Aδ-fiber nociceptors) innervate the same airway region via the SLNs (Canning et al., 2004). This is mimicked in retrograde tracing studies of the same airway segment in which prior unilateral sectioning of an RLN or SLN significantly and specifically reduces the number of retrogradely labeled neurons in the nodose and jugular ganglia, respectively (Mazzone et al., 2009).

In the present study we exploited this differential anatomical arrangement of the afferent innervation to the larynx and bilaterally cut the RLNs in order to generate a physiological preparation in which the majority of the nodose afferent innervation was removed while the majority of the jugular innervation remained intact. In this preparation, almost none of the animals coughed in response to electrical stimulation of the larynx, which should not be interpreted as jugular neurons not evoking cough, given the well describe suppressive effect of anesthesia on cough mediated by nociceptor pathways (Canning et al., 2004). Rather the findings are consistent with the notion that nodose cough receptor innervation (which is not as sensitive to the suppressive effects of anesthesia) was removed (Tsubone et al., 1991; Canning et al., 2004; Mazzone et al., 2005, 2009; Chou et al., 2008). In our preparation, electrical stimulation resulted in intensity and frequency dependent reduction in respiratory output, leading to apnea at higher stimulus parameters suggesting that activation of laryngeal jugular afferents may be inhibitory to respiratory drive. This was accompanied by a modest but consistent fall in blood pressure, but no change in heart rate, perhaps reflective of a diminished respiratory dependent venous return during the apneic response. Compatible with these observations, Chou et al. (2008) previously reported in anesthetized guinea pigs that stimuli selective for jugular vagal afferents evoked respiratory slowing and apnea, while stimuli selective for nodose (mechanoreceptor) vagal afferents evoked cough and/or tachypnea. Of particular interest, blocking the nTS did not alter laryngeal stimulation evoked reductions in respiration, consistent with the notion that nodose afferent pathways were denervated and unlikely involved in this reflex. Indeed, a significant fall in baseline blood pressure following microinjection of muscimol into the nTS confirms the adequacy of the dosing regimen for producing neuronal inhibition. By contrast, laryngeal-evoked respiratory slowing in RLN transected animals was abolished and the hypotension absent when the Pa5 was selectively inhibited by an equivalent dose of muscimol.

We don't know the identity of the vagal afferents mediating the evoked responses in the present study, other than they are of jugular ganglia origin. In guinea pigs, most if not all jugular ganglia afferents are chemically sensitive nociceptors, although both unmyelinated and myelinated subsets exist (Ricco et al., 1996). Consistent with this capsaicin application to the larynx evokes apnea. However, other types of laryngeal afferents also exist, which are perhaps best characterized in larger species such as dogs where subtypes responding to temperature, pressure, “tracheal tug” during breathing or irritants (e.g., hyposmolar solutions) are definable in addition to those responding to classic nociceptor stimuli (reviewed in Sant'Ambrogio et al., 1995). Many of these afferents are nodose-derived mechanoreceptors yet they reach the larynx via the superior laryngeal nerves, suggesting that neither the anatomical pathway for innervation nor the physiological complexity of laryngeal afferent endings in the guinea pig mirrors that of the dog. Accordingly, this makes drawing conclusions about the role of these other subsets in evoked responses difficult. Regardless of the identity of the primary afferents involved, we interpret our current data as strong evidence for a jugular ganglia reflex circuit that can modify respiratory control via synaptic integration in the Pa5.

The present data may also point toward a role for the Pa5 in the regulation of autonomic outflow to the airways and other organs. Thus, chemical stimulation of the laryngeal mucosa can evoke profound bronchoconstrictor responses, which may in part reflect activation of the jugular ganglia-Pa5 circuit. This was not assessed in the present study, but in previous studies in anesthetized guinea pigs we have shown that laryngeal application of capsaicin evokes a bronchoconstrictor response which differs strikingly compared to that when capsaicin is inhaled into lower regions of the airways (Mazzone and Canning, 2002a,b). Indeed, brief (bolus) application of capsaicin onto laryngeal mucosa in anesthetized guinea pigs evokes an initial transient bronchodilation lasting 1–5 min that is replaced by a slowly developing increase in airway smooth muscle tone peaking at 75–100 percent of the maximum attainable response at 30–60 min after the initial bolus capsaicin challenge. When inhaled (in preparations bypassing the larynx) only a bronchoconstriction is seen and the response develops and decays quickly, typically lasting for the duration of the challenge only. Whether these differential responses relate to the nodose and jugular afferent circuits described herein is not known, but conceivably possible. Furthermore, studies from other laboratories have already demonstrated vagal afferent inputs from some baroreceptor afferents terminate in the Pa5 playing a role in cardiovascular control (Junior et al., 2004) and it is intriguing to speculate that these may also be of jugular ganglia origin.

## Conclusions

Our past and present data supports a previously unrecognized role of the Pa5 in mediating laryngeal evoked respiratory reflexes, specifically, those reflexes driven by jugular ganglia derived afferent neurons. Given the somatic nature of the jugular ganglia and the Pa5, it is possible however that this pathway is important for more than just bulbar mediated respiratory reflexes. Strong connectivity of the Pa5 to the somatosensory thalamus (Saxon and Hopkins, 1998; McGovern et al., 2015b) is indicative of jugular neurons providing input to an ascending circuit that may underpin more complex airway sensations. Nevertheless, how these higher order circuits contribute to behaviors associated with airway irritation remain to be elucidated.

## Author contributions

AD conducted experiments, contributed to drafting and editing the manuscript. MF contributed to experimental design and, manuscript editing, SM conceived experiments, contributed to writing and editing of the manuscript, AM conducted experiments, contributed to drafting and editing manuscript. All authors contributed to the interpretation of the data.

### Conflict of interest statement

The authors declare that the research was conducted in the absence of any commercial or financial relationships that could be construed as a potential conflict of interest.
